# Sulfide Production and Microbial Dynamics in the Water Reinjection System from an Offshore Oil-Producing Platform

**DOI:** 10.3390/microorganisms14010038

**Published:** 2025-12-23

**Authors:** Vitória da Silva Pereira Domingues, Maira Paula de Sousa, Vinicius Waldow, Rubens Akamine, Lucy Seldin, Diogo Jurelevicius

**Affiliations:** 1 Instituto de Microbiologia Paulo de Góes, Universidade Federal do Rio de Janeiro, UFRJ, Rio de Janeiro 21941-902, Brazil; vitoria.pereira@micro.ufrj.br (V.d.S.P.D.); lseldin@micro.ufrj.br (L.S.); 2 Centro de Pesquisas Leopoldo Américo Miguez de Mello, CENPES, Rio de Janeiro 21941-915, Brazil

**Keywords:** oil reservoir, secondary oil recovery, sulfate-reducing bacteria, water injection, produced water reinjection

## Abstract

In addition to seawater in the injection header (IH) to enhance oil recovery, oil companies reuse produced water (PW), a byproduct of oil extraction, and implement produced water reinjection systems (PWRI). Although the microorganisms in IH are controlled by biocides, PW is generally treated by flotation to remove oil residues before PWRI. However, IH, PW, and PWRI can be sources of sulfate-reducing bacteria (SRB) related to oil reservoir souring. Here, we evaluated hydrogen sulfide (H_2_S) production in IH, PW, and PWRI, as well as the microbial dynamics (most probable number–MPN, quantitative PCR, and amplicon sequencing), of a Brazilian oil reservoir. Results revealed that the highest average H_2_S concentration occurred in PW samples. However, the dissolved H_2_S threshold concentration of 2 mg L^−1^ was exceeded in 18% of PW and ~16% of PWRI samples, respectively. Although MPN showed no correlation between H_2_S and the number of SRB or total anaerobic heterotrophic bacteria (TAHB), qPCR and microbiome data revealed that the SRB Desulfobacterota was the most abundant in PW and PWRI. Overall, flotation was associated with (i) low microbial control in PW; and (ii) the enrichment of SRB (mainly Desulfobacterota), Thermotogota, and Proteobacteria groups in PWRI.

## 1. Introduction

Secondary oil recovery can enhance petroleum production by up to 50% by increasing the oil reservoir pressure and promoting crude oil displacement into the porous spaces of the reservoir rock [1,2,3,4,5,6]. In general, oil companies use injection water during secondary oil production. One of the consequences of water injection is the production of produced water along with oil extraction at an average ratio of 3:1 of water-to-oil. Produced water is a complex byproduct of oil extraction composed of a mixture of injection water, formation water (the water naturally found in oil reservoirs), and oil [3,7]. The complex composition of produced water includes dissolved minerals and various soluble and insoluble organic and inorganic substances (e.g., hydrocarbons, metals, radioisotopes, and inorganic salts) [7,8].

As secondary oil recovery progresses, the volume of produced water increases [9]. Moreover, because of the different toxic compounds present in produced water, its untreated disposal can be harmful to the marine environment. Oil companies generally resolve this problem by improving produced water treatment (including the removal of dissolved organics) or by implementing produced water reinjection (PWRI). However, before PWRIs, produced water commonly undergoes a flotation process to improve oil removal and prevent clogging of pipes [10,11,12,13]. Flotation uses fine gas bubbles to separate suspended particles and oil droplets from water. In this case, the bubbles cause the oil to rise to the surface, forming a foam that is skimmed off from the produced water. In this way, flotation is effective for removing oil, grease, and volatile organics from produced water, contributing to chemical control of the composition of the produced water [7,13].

One of the main problems faced by oil companies during secondary recovery is the control of microbial metabolism, especially that of sulfate-reducing bacteria (SRB). In oil reservoirs, SRBs are related to the production of high amounts of H_2_S during the dissimilatory sulfate reduction process. High concentrations of H_2_S can result in oil reservoir souring, and if the H_2_S threshold is exceeded, oil production from an acid oil reservoir can be promptly interrupted [14,15]. In addition, SRB biofilms and H_2_S can accelerate corrosion (microbiologically influenced corrosion) and lead to the formation of precipitates such as iron sulfide. Taken together, H_2_S production by the SRB negatively impacts oil recovery productivity [3,16,17,18], forcing the drilling of new oil reservoirs and increasing the risk of environmental problems [19,20].

Oil reservoirs offer a broad range of niches for bacteria and archaea, such as sulfate, nitrate, and iron reducers, fermenters, acetogens, and methanogens [21]. In waterflooded oil reservoirs, the microbial community is a mixture of microorganisms autochthonous to the oil reservoir plus allochthonous microorganisms that are introduced into the oil reservoir with the injection water [22]. However, recent studies have highlighted the increased presence of sulfate-reducing bacteria (SRB) and increased sulfate availability in oil reservoirs after waterflooding [22,23]. Although water injection systems of oil-producing platforms involve different steps to treat the injection water (chlorination, filtration, deaeration, sulfate removal, and biocide treatments), most of the produced water is treated mainly by flotation systems before being used as PWRI in the water reinjection manifold system. Therefore, we hypothesize that (i) PW can promote the contamination of PWRI with sulfide-producing bacteria (SPB) and/or (ii) flotation systems can reduce the microbial load and SRB abundance in PWRI. To test these hypotheses, here we coupled chemical (H_2_S-production) and microbial analyses to evaluate the first long-term monitoring of IH–PW–PWRI systems from a Brazilian offshore platform. Mapping H_2_S production and the microbial composition of IH–PW–PWRI systems could provide valuable insights for developing effective strategies to mitigate microbial contamination and improve secondary oil recovery.

## 2. Materials and Methods

### 2.1. Field and Sampling Sites for Chemical and Culture-Based Microbiological Analyses

The oil-producing platform used in this study is located in the Campos Basin (Atlantic Ocean, Rio de Janeiro, Brazil), and its activity started in January 2009. The platform produces oil from a reservoir located 3 km below sea level, and the temperature of the oil reservoir is approximately 80 °C. For secondary oil recovery, the platform primarily uses a seawater flooding system where the seawater is previously filtered (5 μm), deoxygenated by gas stripping, desulfated by nanofiltration, and finally treated in a seawater injection header (IH) with approximately 300 ppm Tetrakis hydroxymethyl phosphonium sulfate (THPS) every 3 days. Additionally, the platform implemented produced water reinjection (PWRI). For PWRI, the suspended particles and the oil droplets from produced water (PW) are separated using fine gas bubbles in a flotation process. Oil-free produced water (PWRI) is then injected into the oil reservoir via a water reinjection header.

For chemical and culture-based microbiological analyses, three kinds of samples were periodically collected from January 2022 to April 2024 (Table S1): injection water (IH), produced water (PW), and reinjection produced water (PWRI). The IH samples (*n* = 22) were all obtained from the injection water manifold system after seawater treatment, as described above. PWs (*n* = 90) were sampled directly from the oil-collecting pipes, whereas PWRIs were sampled after the flotation process in the water reinjection manifold system (*n* = 460). All the samples were kept anoxic at −20 °C. The samples were transported to the laboratory and immediately processed for microbiological analyses.

### 2.2. Chemical Analysis

The analytical method used in this study was based on the standard method 4500-S^2^ described by the American Public Health Association [24]. Samples were taken by collecting the needed aliquot in a beaker just before performing the analysis. Sulfide analysis was carried out by iodometric titration, as described in detail in Method 4500-S^2^ F. Given the non-normal distribution, statistical differences in sulfide concentrations in PW, PWRI, and IH were determined based on the non-parametric Mann–Whitney test (*p* < 0.05).

### 2.3. Abundances of Sulfate-Reducing Bacteria (SRB) and Total Anaerobic Heterotrophic Bacteria (TAHB)

The abundances of sulfate-reducing bacteria (SRB) and total anaerobic heterotrophic bacteria (TAHB) in PW, PWRI, and IH were quantified by the multiple tube technique (most probable number, MPN) using Postgate Medium E [25] and TAHB medium [26], respectively, as described in Sakamoto et al. [27]. For MPN determination, SRB growth was considered positive when the presence of a black precipitate of iron sulfide was observed. For TAHB, positivity is indicated by turbidity and discoloration in the culture medium. Differences in the non-normal MPN values between PW, PWRI, and IH samples were analyzed via the non-parametric Mann-Whitney test (*p* < 0.05).

### 2.4. DNA Extraction for Molecular-Based Microbiome Analyses

The samples for microbiome analysis were collected in October 2022, since at this time the first observed increase in sulfide in PW (in August 2022) was followed by an increase in sulfide in PWRI (October 2022). Then, for microbiome analysis, at least 250 mL of water samples were collected from PW (*n* = 3) and PWRI (*n* = 6) in October 2022. Each water sample collected was immediately filtered through a 0.22 µm Millipore membrane. Each of the membranes containing the microbial cells was subsequently used for DNA extraction through the FastDNA Kit following the manufacturer’s recommendations (QBIOgene, Carlsbad, CA, USA). The quantification of the extracted DNA from each sample was performed via a NanoDrop 1000 spectrophotometer (Thermo Scientific, Suwanee, GA, USA).

### 2.5. Real-Time Quantitative PCR (qPCR)

The prokaryotic abundance in the PW and PWRI samples was quantified by qPCR targeting the *16S rRNA* gene. The reactions were performed with 2 μL of the extracted DNA using PowerUp SYBR Green Master Mix (Applied Biosystems, Waltham, MA, USA) in a final reaction volume of 12 μL. The primers used were 357F (CCTACGGGAGGCAGCAG) and 529R (ATTACCGCGGCTGCTGG) [28]. Quantification was performed in triplicate for each sample on a QuantStudio3 thermocycler (Thermo Fisher). Melting curve analysis was performed with a gradual increase in temperature (0.15 °C/s) from 60 °C to 95 °C to confirm the specificity of the amplification. Standard curves were obtained using 10-fold serial dilutions (10^7^ to 10^1^ gene copies per mL) of a plasmid containing cloned *16S rRNA* gene fragments prepared in nuclease-free water. The amplification data were then analyzed using QuantStudio Design and Analysis Software 3 System. The normality of the distribution and homoscedasticity among treatments for all qPCR values were assessed via the Shapiro-Wilk test prior to pairwise comparisons. Differences in the qPCR values between the PW and PWRI samples were analyzed using the Kruskal-Wallis test, with statistical significance set at *p* < 0.05.

### 2.6. Microbiome Analysis on the Basis of 16S rRNA Gene Sequences

To characterize the microbial community present in PW and PWRI, the *16S rRNA* gene sequences were sequenced via the MiSeq platform (Illumina, San Diego, CA, USA). The primers used for sequencing were 341F (CCTAYGGGRBGCASCAG) and 806R (GACTACNNGGGTATCTAAT), which target the V3–V4 region of the 16S rRNA gene [29]. The *16S rRNA* gene sequences were subsequently analyzed using the QIIME2 pipeline (version 2022.2). In this process, the sequences were demultiplexed as described by Bolyen et al. [30]. Quality control was then applied using the Deblur algorithm, which eliminates chimeric and low-quality sequences. High-quality sequences that passed the filtering step were subsequently grouped into amplicon sequence variants (ASVs), following the method outlined by Amir et al. [31]. To assign taxonomic annotations to the obtained ASVs, the SILVA (v138.1) database [32] was used.

The alpha diversity of the bacterial communities present in the PW and PWRI samples was estimated using the observed ASV counts, the Shannon diversity index [33], and Faith’s phylogenetic diversity [34]. The alpha diversity metrics of all the samples were compared via the Kruskal-Wallis test. In addition, beta diversity was compared using weighted and unweighted UniFrac distance significance analysis and Principal Component Analysis (PCoA). The beta diversity results were statistically compared via PERMANOVA [35].

To estimate the absolute abundances of bacteria present in each PW and PWRI sample, the values obtained from bacterial qPCR were considered to represent the number of bacterial cells in each sample, as suggested by Morton et al. [36]. The values of the relative abundance of each bacterial phylum, class, and genus (based on sequencing data) were considered the fraction of the represented groups in each sample (as described in Jian et al. [37]). Thus, the relative abundances were translated into estimated absolute abundances, assuming a roughly similar *16S rRNA* gene copy number across taxa, by multiplying the relative abundance of each taxon by the total bacterial/archaeal abundances (qPCR results) in the sample. Statistical differences in estimated absolute abundances between PW and PWRI samples were analyzed via the Kruskal-Wallis test (*p* < 0.05).

## 3. Results

### 3.1. Analysis of H_2_S in the Oil Reservoir

**Figure 1:**
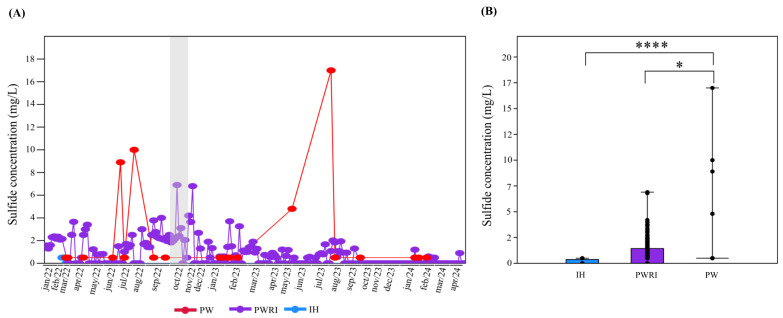
(**A**) Temporal visualization of sulfide concentrations in samples from produced water (PW-red), produced water reinjection (PWRI-purple), and the injection header (IH-blue). The area marked in gray represents the period used for the microbiome analysis (October 2022). (**B**) Box plot of sulfide concentrations in produced water (PW), produced water reinjection (PWRI), and the injection header (IH). (*) and (****) indicate significant differences according to the Mann-Whitney test (*p* < 0.05 and *p* < 0.0001, respectively).

The H_2_S concentrations in the IH, PW, and PWRI samples were analyzed for 27 months (Figure 1A,B). Although H_2_S concentrations were less than 0.5 mg L^−1^ in all IH samples analyzed, in both PW and PWRI, the concentration of H_2_S varied during the analyzed months (Figure 1A,B). In PW, the H_2_S concentration varied from 0.5 mg L^−1^ to 17 mg L^−1^ (detected in samples collected from July 2023), whereas in PWRI samples, the H_2_S concentration varied from 0 mg L^−1^ to 6.9 mg L^−1^ (detected in samples collected from October 2022) (Figure 1A). Furthermore, the average H_2_S concentration was significantly greater in the PW samples (average of 2.4 mg L^−1^) than in the PWRI (0.9 mg L^−1^) and IH (below 0.5 mg L^−1^) samples (*p* < 0.05) (Figure 1B).

### 3.2. Abundances of SRB and TAHB in PW and PWRI Samples

**Figure 2:**
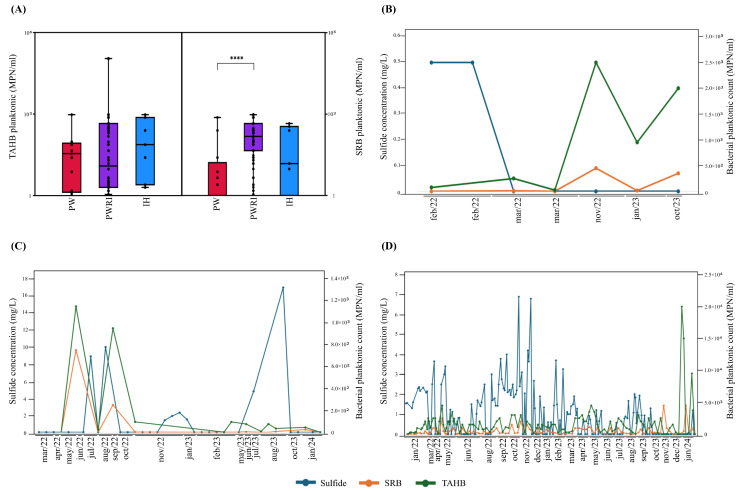
Box plots of (**A**) total anaerobic heterotrophic bacteria (TAHB) planktonic counts and sulfate-reducing bacteria (SRB) in produced water (PW), produced water reinjection (PWRI), and injection header (IH) samples. (****) Indicates significant differences according to the Mann-Whitney test (*p* < 0.0001). Temporal visualization of sulfide concentration (blue) and sulphate-reducing bacteria (SRB-orange) and total anaerobic heterotrophic bacteria (TAHB-green) count in (**B**) injection header (IH), (**C**) produced water (PW), and (**D**) produced water reinjection (PWRI) samples.

The analysis of the cultivable fractions of SRB and TAHB in the PW, PWRI, and IH samples was performed via MPN estimation. The results revealed no differences in the average TAHB counts between the PW, PWRI, and IH samples (Figure 2A). On the other hand, although no significant differences were observed between the SRB cell counts detected in the IH samples and those detected in the PW and PWRI samples, the MPN results revealed that the SRB cell count was significantly greater in the PWRI samples than in the PW samples (Figure 2A; *p* < 0.0001).

However, as observed for the H_2_S concentration, the MPNs of SRB and TAHB varied during the analyzed months in the samples collected from IH (Figure 2B), PW (Figure 2C), and PWRI (Figure 2D). Furthermore, Spearman linear correlation analysis was used to evaluate the correlation between the sulfide concentration and the SRB or TAHB cell count in IH, PW, and PWRI. The results revealed no significant correlation between the sulfide concentration and the SRB or TAHB cell count in the IH, PW, and PWRI samples (*p* > 0.05).

### 3.3. Total 16S rRNA Abundance and Alpha and Beta Diversity Analyses of the Prokaryotic Communities Present in the PW and PWRI Samples

**Figure 3:**
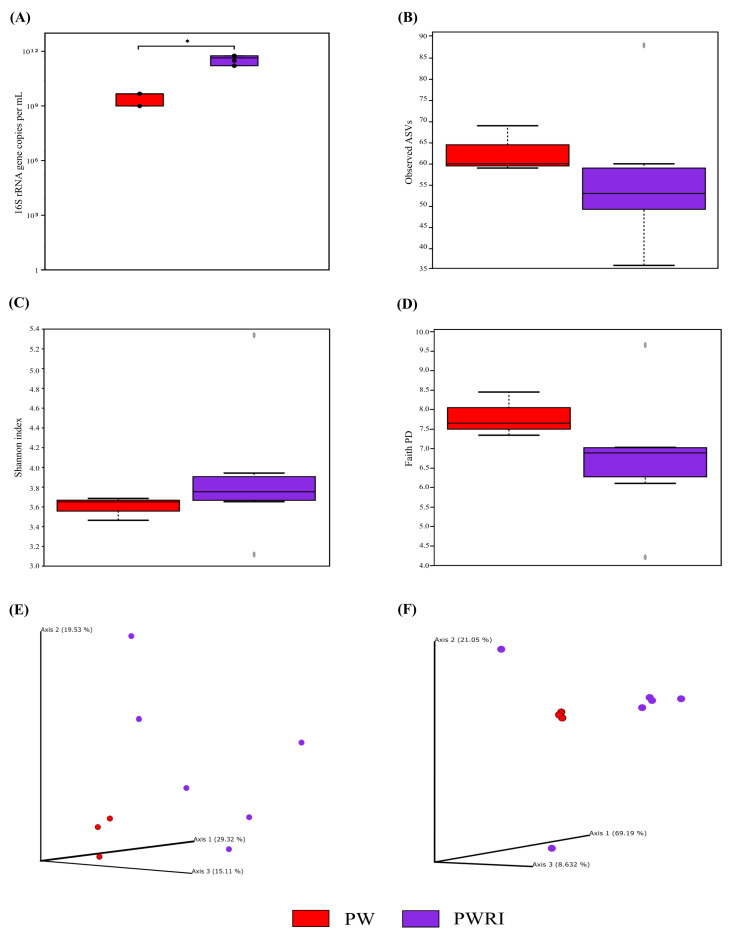
(**A**) Total abundance graph of prokaryotes in produced water (PW-red) and produced water reinjection (PWRI-purple) samples. (*) Indicates significant differences according to the Kruskal-Wallis test (*p* < 0.05). Alpha diversity plots based on (**B**) the observed ASV, (**C**) Shannon index, and (**D**) the Faith-PD index for the PW and PWRI samples. (**E**,**F**) Beta diversity analysis via principal coordinate analysis (PCoA) based on (**E**) unweighted UniFrac and (**F**) weighted UniFrac distances for amplicon sequence variants (ASVs) in produced water (red points) and produced water reinjection (purple points) samples. Points outside the whiskers represent outliers, plotted as individual dots according to the 1.5 × IQR criterion.

To determine the abundance, diversity, and composition of the prokaryotic groups related to the presence of high concentrations of H_2_S in PW and PWRI, different samples collected in October 2022 (when the H_2_S concentration reached 6.9 mg L^−1^ in PWRI) were selected for analysis on the basis of methods independent of microbial culture. First, the results of the qPCR analysis showed that the abundance of prokaryotic cells was greater in all the PWRI samples than in the PW samples (*p* < 0.05). The results revealed that the average number of *16S rRNA* gene copies in 1 mL in PWRI (8.11 × 10^11^) was 38 times greater than that in PW (2.11 × 10^10^) (Figure 3A).

However, alpha-diversity analyses revealed no significant differences in the microbial diversity present in the PW and PWRI samples (Figure 3B–D), although the average observed ASVs and the Faith-PD index were greater in the PW samples than in the PWRI samples (Figure 3B,D), and the Shannon index values were greater in the PWRI samples than in the PW samples (Figure 3C). The same results were observed when the structure and composition of the prokaryotic communities present in the PW and PWRI samples were compared (Figure 3E,F). Principal Coordinate Analysis (PCoA) and beta diversity significance analyses based on weighted and unweighted UniFrac metrics revealed no significant differences between the composition and distribution of the microbial communities present in the PW and PWRI samples (*p* > 0.05) (Figure 3E,F, respectively).

### 3.4. Microbiome Composition in PW and PWRI Samples

**Figure 4:**
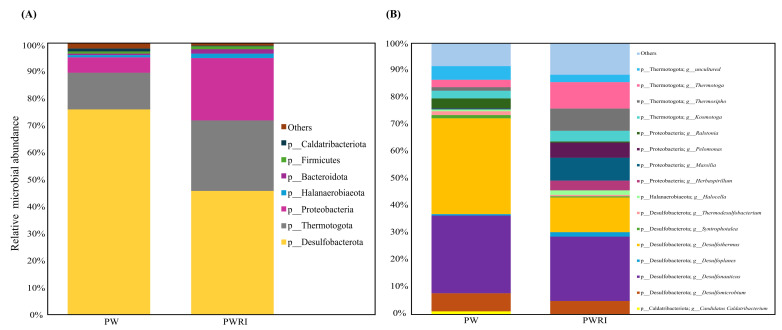
Relative abundance of the main prokaryotic phyla (**A**) and of the main prokaryotic genera (**B**) present in the PW and PWRI samples.

**Figure 5:**
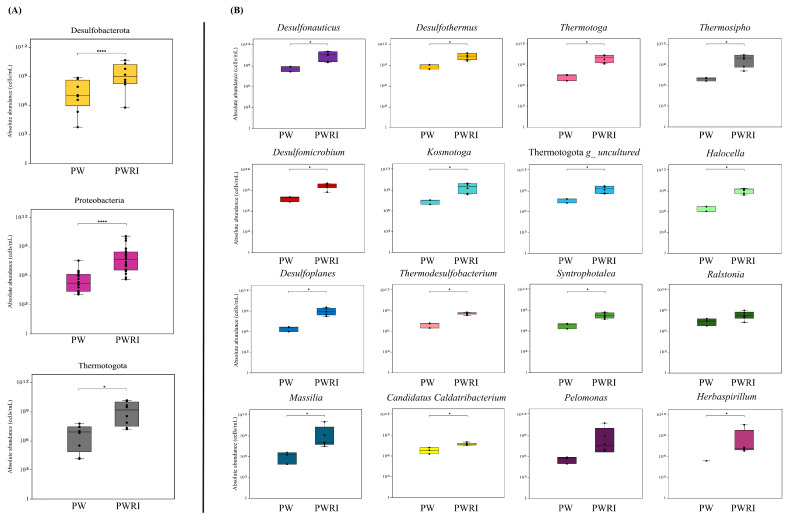
Absolute abundance of the main prokaryotic (**A**) phyla and (**B**) genera determined on the basis of the qPCR values obtained for PW and PWRI samples. (*) and (****) indicate significant differences according to the Kruskal-Wallis test (*p* < 0.05 and *p* < 0.0001, respectively).

In total, 21 different phyla (mainly Desulfobacterota, Proteobacteria, and Thermotogota) were detected in the PW and PWRI samples, and 73 and 87 prokaryotic genera were detected in the PW and PWRI samples, respectively (Figure 4A,B; Figure 5A,B; Table S2). The relative abundance and the estimated absolute abundance (normalized on the basis of qPCR values) of the main microbial phyla and genera observed in the PW and PWRI are shown in Figure 4A and Figure 5A. The results revealed that the bacterial phylum Desulfobacterota was the most abundant phylum in the PW and PWRI samples (Figure 4A). In PW, Desulfobacterota represented 75% of the relative abundance of the microbial community (Figure 4A) and accounted for an estimated absolute abundance of 1.59 × 10^10^ cells per mL (Figure 5A). Considering the PWRI samples, Desulfobacterota represented 45% of the relative abundance of the microbial community and accounted for an estimated absolute abundance of 3.66 × 10^11^ cells per mL (Figure 4A and Figure 5A, respectively). The second most abundant bacterial phylum present in PW and in PWRI was the thermophilic and hyperthermophilic phylum Thermotogota. In this case, the relative abundances of Thermotogota were 13% and 26% in the PW and PWRI samples, respectively, accounting for absolute abundances of 2.81 × 10^9^ and 2.1 × 10^11^ in the PW and PWRI samples, respectively (Figure 4A and Figure 5A, respectively). Interestingly, the phylum Proteobacteria represented ~6% of the relative abundance of microbial communities present in the PW samples, and 23% of the relative abundance of microbial communities present in the PWRI samples. The estimated absolute abundance of the Proteobacteria phylum was 1.23 × 10^9^ cells/mL in the PW sample and 1.86 × 10^11^ cells/mL in the PWRI samples (Figure 4A and Figure 5A, respectively).

Furthermore, the results revealed that different bacterial groups were enriched in the PWRI samples compared with their abundance in the PW samples. For example, the absolute abundance of the Proteobacteria phylum was 151 times greater in the PWRI samples than in the PW samples and represented the most enriched bacterial phyla in the PWRI samples. The same results were observed for Thermotogota and Desulfobacterota. The absolute abundance of the phylum Thermotogota was 75 times greater in the PWRI samples than in the PW samples. Although the relative abundance of Desulfobacterota decreased from 75% in PW to 45% in PWRI, the absolute abundance of the Desulfobacterota phylum was still 23 times greater in PWRI than in PW.

The relative abundance and the estimated absolute abundance of the main microbial genera observed in the PW and PWRI are shown in Figure 4B and Figure 5B and Table S2. The results revealed that 92% of the prokaryotic genera present in the PW samples were also detected in the PWRI samples. However, 21 bacterial genera (including 11 genera from the Proteobacteria phylum, 5 from Bacteroidota, and others from Actinobacteriota, Deinococcota, Fibrobacterota, Firmicutes, and Verrucomicrobiota) were exclusively detected in the PWRI samples (Table S2). Although these genera did not represent the most abundant groups in PWRI, they accounted for 2.47% (2.00 × 10^10^) of the estimated bacterial cells present in PWRI samples.

Considering the bacterial genera, the SRB-related genera *Desulfothermus* (~36%) and *Desulfonauticus* (29%) were the most abundant bacterial genera in the PW samples, whereas *Desulfonauticus* (24%) and *Desulfothermus* (13%) were predominant in the PWRI samples (Figure 4B). Interestingly, the dominant bacterial groups differed between the PW and PWRI samples. In addition to *Desulfothermus* and *Desulfonauticus*, the other abundant genera in the PW samples were related to *Desulfomicrobium* (6.8%), an uncultured genus of the Thermotogota phylum (5.1%), *Ralstonia* (3.5%), *Kosmotoga* (2.9%), and *Thermotoga* (2.8%), among others (Figure 4B). On the other hand, in PWRI, the other dominant bacterial groups observed were related to the genera *Thermotoga* (9.9%), *Massilia* (8.5%), *Thermosipho* (8.4%), *Pelomonas* (5.5%), and *Desulfomicrobium* (4.8%), among others (Figure 4B).

Absolute abundance estimation revealed that the SRB-related genera *Desulfothermus* and *Desulfonauticus* represented 7.60 × 10^9^ and 6.15 × 10^9^ cells per mL of PW samples, respectively, and 1.06 × 10^11^ and 1.96 × 10^11^ cells per mL of PWRI samples, respectively (Figure 5B). These results revealed that *Desulfothermus* and *Desulfonauticus* were enriched 14- and 31-fold in PWRI, respectively, compared with PW (Figure 5B). Bacteria from the phylum Thermotoga were also enriched in the PWRI samples. For example, the estimated absolute abundances of the genera *Thermosipho* and *Thermotoga* were 2.78 × 10^8^ and 5.92 × 10^8^ cells per mL, respectively, in the PW samples and 6.85 × 10^10^ and 8.02 × 10^10^ cells per mL, respectively, in the PWRI samples (Figure 5B). The absolute abundances of *Thermosipho* and *Thermotoga* in the PWRI samples were 235 and 135 times greater than those in the PW samples, respectively.

However, considering the most abundant genera observed in PW and PWRI, the results revealed that *Herbaspirillum* was the most enriched bacterial genus in PWRI samples (the absolute abundance of *Herbaspirillum* was 5560 times greater in PWRI than in PW samples), followed by bacteria related to the genera *Pelomonas* and *Massilia,* which were 1730 and 905 times greater, respectively, in PWRI than in PW (Figure 5B). Interestingly, *Herbaspirillum, Pelomonas,* and *Massilia* represented only 0.03%, 0.12% and 0.36%, respectively, of the relative abundance of the bacterial community present in the PW samples (Figure 4B).

## 4. Discussion

### 4.1. Biogenic Sulfide Production Dynamics

Biogenic hydrogen sulfide production in oil reservoirs postflooding is a significant economic issue in the oil and gas industry and can be stimulated by the reinjection of produced water [38]. Here, by analyzing the water injection system (IH), produced water system (PW), and produced water reinjection system (PWRI) of one oil-producing platform from the Campos Basin, Brazil, we showed that the highest concentration of H_2_S was detected in PW samples (17 mg L^−1^ detected in samples collected from July 2023). In addition, the average sulfide concentration detected in PW was significantly greater than the average sulfide concentration observed in the IH and PWRI samples. The sulfide concentration in the PWRI samples varied during the analyzed months and reached 6.9 mg L^−1^ in the samples collected from October 2022. On the other hand, consistently low sulfide concentrations were observed under IH (the highest sulfide concentration detected under IH was 0.5 mg L^−1^). Considering that biogenic souring is often associated with SRB activity, the low sulfide levels detected at IH are consistent with the reduced presence of SRBs in seawater [39]. In addition, seawater injection systems are constantly treated with biocides such as THPS, which potentially reduce microbial abundance under IH [23,40,41,42]. However, despite all seawater treatments, seawater injection can still favor souring because of sulfate input, which modulates microbial metabolism in oil reservoirs [43].

Furthermore, our results suggest that the majority of sulfide production occurs in oil reservoirs during water-flooded oil production, promoting a high concentration of H_2_S in PW samples. However, our results revealed that sulfide was also produced in PWRI samples after the flotation treatment of PW. Although flotation is effective for the chemical control of the composition of produced water, which mainly removes oil and grease from PW [7,13], its effect on sulfide production is underexplored. We hypothesize that (i) the contamination of PWRI with sulfide-producing bacteria (SPB) present in PW or (ii) the enrichment of SPB unrelated to those present in PW samples by the flotation process increases sulfide production in the PWRI system. If one of these hypotheses is correct, the direct consequence would be SPB contamination in all the steps after the PWRI system, which would increase the abundance of SPB in oil reservoirs and potentially cause a constant increase in sulfide production in oil reservoirs.

As shown by Eden et al. [44] and da Silva & Cintra [45], H_2_S values above 2 mg L^−1^ in oil reservoirs are consistent with reservoir souring. The results obtained from the temporal analysis revealed that the H_2_S dissolved threshold concentration of 2 mg L^−1^ exceeded 18% of the analyzed PW samples and ~16% of the analyzed PWRI samples. These results suggested that, despite our hypothesis, there was no sustained upward trend in H_2_S detected in the oil reservoirs (PW samples) observed during the monitoring period, despite the reinjection of PWRI. In contrast, peaks in sulfide production were observed during the analyzed months in both the PW and PWRI samples.

Furthermore, the fluctuations in H_2_S concentrations in the PW and PWRI samples suggested complex dynamics of microbial processes in oil reservoirs and in PWRI systems during oil production. As highlighted by Jurelevicius et al. [23], H_2_S concentrations in the oil field environment can exhibit notable variability over time and are likely influenced by multiple factors. Hagar et al. [46] reported that changes in reservoir environmental parameters such as nutrient availability (e.g., nitrogen and phosphorus), temperature, pressure, pH, salinity, sulfate concentration (serving as electron acceptors), organic compounds, and hydrogen (acting as electron donors) can affect H_2_S production in oil reservoirs. In addition, H_2_S production can be influenced by the efficiency of microbial control strategies during secondary oil recovery, which impacts both H_2_S levels and microbial community composition in oil reservoirs [23,47,48].

### 4.2. Total 16S rRNA Abundance in Produced Water Systems, Abundance of Planktonic SRB and TAHB, and Sulfide Correlation

Generally, H_2_S production in oil reservoirs is stimulated when environmental parameters are aligned with conditions that enable the maximum growth rate of SRBs, which depends on the specific SRB groups present [49]. Additionally, both planktonic and biofilm-associated SRB can contribute to sulfate reduction and H_2_S production [50,51]. To evaluate the microbial community related to H_2_S production, we first used MPN estimation of planktonic SRB and TAHB in the PW, PWRI, and IH samples. Overall, the MPN estimation revealed low planktonic counts for SRB and TAHB in the IH, PW, and PWRI samples. Although the highest average SRB content was obtained from the PWRI samples, compared with the PW samples, the results revealed no correlation between the planktonic TAHB and SRB count estimation results and the sulfide levels detected in the PW, PWRI, and IH samples.

In addition, the choice of method strongly affects the abundance estimates obtained for planktonic communities. Notably, the limitations of the MPN technique in detecting SRB groups have been highlighted in previous oil field studies [18,52,53]. Senthilmurugan et al. [52] reported relevant differences between the MPN and qPCR methods in the estimation of total prokaryote and SRB abundance. In addition, Zhu et al. [53] reported that the qPCR results for SRB abundance were 2 to 5 log higher than those obtained by MPN. Additionally, as is well known, many SRBs that are found as biofilm-associated populations can support sulfate reduction even when the number of planktonic cells in the bulk water is low [50,51]. These data may explain why our results revealed low planktonic SRB abundances, mainly in the PW and PWRI samples, even when the sulfide concentration surpassed 8.5 times the threshold of sulfide levels in the PW samples and ~3.5 times the sulfide level in the PWRI samples.

Consistent with the results obtained by Senthilmurugan et al. [52] and Zhu et al. [53], the qPCR analysis performed here with PW and PWRI samples collected in October 2022 (the period where the highest concentration of sulfide was observed in PW and in PWRI samples) revealed the presence of 8.11 × 10^11^ and 2.11 × 10^10^ prokaryotic cells per mL of PWRI and PW samples, respectively (values 2 to 5 log higher than those obtained by MPN). Furthermore, these results revealed that the number of prokaryotic cells was 38 times greater in the PWRI samples than in the PW samples (*p* < 0.05). On the basis of these results, we suggest that the first effect of PW flotation treatment is to increase the prokaryotic cell abundance in PWRI systems.

### 4.3. Diversity, Composition, and Effects of Flotation Treatment on the Prokaryotic Communities of PW and PWRI Samples

H_2_S production is more dependent on which prokaryotic groups are present than on the prokaryotic abundance. As mentioned previously, the presence of SRB in PW and the contamination or enrichment of SRB by flotation treatment in PWRI could explain the high concentration of H_2_S detected in both PW and PWRI. Our microbiome results revealed that the alpha and beta diversities of the prokaryotic community present in the PW and PWRI samples were not significantly different (*p* > 0.05), and 91% of the prokaryotic genera detected in the PW samples were also detected in the PWRI samples. Although the microbiome analysis was based only on samples collected in October 2022 (from a 27-month monitoring period), these results suggested that (i) flotation treatment had a limited effect on controlling the prokaryotic community present in PW samples and that (ii) PWRI systems are contaminated with most of the prokaryotic community present in oil reservoirs (PW samples).

Our data also revealed a modulation of bacterial groups before and after flotation treatment (in PW and PWRI, respectively), providing insights into how flotation treatment affects the prokaryotic community in PWRI systems. For example, before flotation, the main Desulfobacterota found in PW were related to *Desulfothermus* (~36%) and *Desulfonauticus* (29%), whereas *Desulfonauticus* (24%) and *Desulfothermus* (13%) were predominant after flotation in PWRI samples. Both *Desulfothermus* and *Desulfonauticus* have been described as hyperthermophilic SRB [54,55,56]. Sokolova et al. [57], for example, reported the relationship between *Desulfonauticus* and H_2_S production in high-temperature oil fields in the Republic of Kazakhstan. In addition, Jurelevicius et al. [23] reported the presence of bacteria related to *Desulfonauticus* in the microbial community of Brazilian high-temperature oil fields. Although different species of SRB related to the *Desulfothermus* genus have been described, this genus is less frequently detected in produced water and oil field samples. Interestingly, some *Desulfothermus* species, such as *Desulfothermus naphthae*, grow with alkanes and long-chain fatty acids but not with sugars, amino acids, dicarboxylic acids, or complex substrates such as yeast extract [58].

In addition, our results revealed that the main effect of the flotation of PW was the enrichment of bacteria from the Proteobacteria and Thermotogota phyla in the PWRI samples. Specifically, our results revealed that the absolute abundance of Proteobacteria and Thermotogota was significantly enriched in PWRI samples compared with PW samples. Proteobacteria and Thermotogota are typically encountered in oil reservoirs [59,60,61,62] and include representative groups involved in fermentation processes, nitrate reduction, and hydrocarbon oxidation, among other microbial processes [63,64]. For example, the hyperthermophilic genera *Thermosipho* and *Thermotoga*, representatives of the Thermotogota phylum, were detected in both PW and PWRI samples and were enriched 235 and 135 times, respectively, in PWRI samples. These bacteria are recognized as carbohydrate fermenters that produce volatile fatty acids and hydrogen as end products [59,60]. In oil reservoirs, the metabolic byproducts produced by bacteria from the Thermotogota phylum are well known to support the metabolism of different microorganisms, including SRB [59,60,65]. In addition to fermentation, *Thermosipho* can actively participate in the sulfur cycle because of its potential to reduce thiosulfate and sulfur to sulfide [57,60].

Interestingly, the most enriched bacterial genera in the PWRI samples were related to the Proteobacteria phylum. For example, of the 21 prokaryotic genera detected exclusively in the PWRI samples, 11 were related to the Proteobacteria phylum. In addition, our results revealed that the most enriched bacterial genera after the flotation process were related to the Proteobacteria-containing genera *Herbaspirillum*, *Pelomonas,* and *Massilia.* These bacterial genera, which together represented less than 0.2% of the relative abundance of the prokaryotic community present in the PW samples, were mainly introduced into PWRI by the flotation process. As also shown by Ren et al. [66], bacteria from genera such as *Pelomonas*, *Herbaspirillum,* or *Massilia* have rarely been detected in oil reservoirs or in produced water systems.

For example, the *Herbaspirillum* genus has been studied more as a plant growth-promoting bacterium, although these anaerobic or facultative anaerobic bacteria represent low-abundance bacteria that are significantly associated with CO_2_ injection and present in formation water from the Olla Oil Field in LaSalle Parish, LA, USA [67]. The genus *Pelomonas* has also been detected as a low-abundance bacterial genus in produced water from the high-temperature Shengli oil field, which is located in China [66]. One of the hypotheses for the enrichment of these prokaryotic genera in PWRI after the flotation process is the previously described ability of bacteria from these genera to degrade different petroleum hydrocarbons [68,69,70,71,72,73]. However, although the consequences of the presence of these Proteobacteria genera in oil reservoirs are still unknown, these bacteria have an improbable influence on sulfide production in PWRI and in oil reservoirs.

## 5. Conclusions

The results shown here highlight the dynamics of H_2_S production in a Brazilian oil-producing platform. Although most of the detected H_2_S production occurs in oil reservoirs (as detected in PW samples), our results revealed H_2_S production in PWRI systems. Overall, our results demonstrated that PWRI, downstream of flotation, exhibited (i) markedly higher prokaryotic abundance and (ii) the enrichment of SRB (mainly Desulfobacterota), Thermotogota, and Proteobacteria groups. These results suggested that the flotation step as implemented does not reduce, and may favor, the survival and/or the enrichment of bacteria delivered from PW. Furthermore, the results explained the high H_2_S levels detected in both PW and PWRI systems. Nevertheless, these findings highlight the need to improve microbial control in oil-producing platforms (such as with the use of different biocides in PWRI systems) and underscore the critical role of SRB and their potential to compromise oil reservoir integrity.

## Data Availability

The original contributions presented in the study are included in the article/Supplementary Material, NCBI Accession PRJNA1363945, and further inquiries can be directed to the corresponding author.

## Supplementary Materials

The following supporting information can be downloaded at https://www.mdpi.com/article/10.3390/microorganisms14010038/s1. Table S1: Temporal monitoring of sulfide and bacterial populations in injection water, produced water, and reinjection water samples (2022–2024); Table S2: Microbiome composition and estimated absolute abundances of PW and PWRI samples.

## Abbreviations

The following abbreviations are used in this manuscript:

IH	Injection header
PW	Produced water
PWRI	Produced water reinjection
SRB	Sulfate-reducing bacteria
TAHB	Total anaerobic heterotrophic bacteria
H_2_S	Hydrogen sulfide
MPN	Most probable number
qPCR	Quantitative polymerase chain reaction
ASV	Amplicon sequence variant
PCoA	Principal coordinate analysis
PD	Phylogenetic diversity
SPB	Sulfide-producing bacteria
THPS	Tetrakis hydroxymethyl phosphonium sulfate
DNA	Deoxyribonucleic acid
16S rRNA	16S ribosomal RNA
